# Guidelines and mHealth to Improve Quality of Hypertension and Type 2 Diabetes Care for Vulnerable Populations in Lebanon: Longitudinal Cohort Study

**DOI:** 10.2196/mhealth.7745

**Published:** 2017-10-18

**Authors:** Shannon Doocy, Kenneth E Paik, Emily Lyles, Hok Hei Tam, Zeina Fahed, Eric Winkler, Kaisa Kontunen, Abdalla Mkanna, Gilbert Burnham

**Affiliations:** ^1^ Johns Hopkins Bloomberg School of Public Health Baltimore, MD United States; ^2^ Sana mHealth Group Massachusetts Institute of Technology Cambridge, MA United States; ^3^ Sana mHealth Group Department of Chemical Engineering Massachusetts Institute of Technology Cambridge, MA United States; ^4^ International Organization for Migration Beirut Lebanon

**Keywords:** mHealth, hypertension, diabetes mellitus, chronic disease, Lebanon, Syria, refugees

## Abstract

**Background:**

Given the protracted nature of the crisis in Syria, the large noncommunicable disease (NCD) caseload of Syrian refugees and host Lebanese, and the high costs of providing NCD care, the implications for Lebanon’s health system are vast.

**Objective:**

The aim of this study was to evaluate the effectiveness of treatment guidelines and a mobile health (mHealth) app on quality of care and health outcomes in primary care settings in Lebanon.

**Methods:**

A longitudinal cohort study was implemented from January 2015 to August 2016 to evaluate the effectiveness of treatment guidelines and an mHealth app on quality of care and health outcomes for Syrian and Lebanese patients in Lebanese primary health care (PHC) facilities.

**Results:**

Compared with baseline record extraction, recording of blood pressure (BP) readings (−11.4%, *P*<.001) and blood sugar measurements (−6.9%, *P*=.03) significantly decreased following the implementation of treatment guidelines. Recording of BP readings also decreased after the mHealth phase as compared with baseline (−8.4%, *P*=.001); however, recording of body mass index (BMI) reporting increased at the end of the mHealth phase from baseline (8.1%, *P*<.001) and the guidelines phase (7.7%, *P*<.001). There were a great proportion of patients for whom blood sugar, BP, weight, height, and BMI were recorded using the tablet compared with in paper records; however, only differences in BMI were statistically significant (31.6% higher in app data as compared with paper records; *P*<.001). Data extracted from the mHealth app showed that a higher proportion of providers offered lifestyle counseling compared with the counseling reported in patients’ paper records (health diet counseling; 77.3% in app data vs 8.8% in paper records, *P*<.001 and physical activity counseling and 59.7% in app vs 7.1% in paper records, *P*<.001). There were statistically significant increases in all four measures of patient-provider interaction across study phases. Provider inquiry of medical history increased by 16.6% from baseline following guideline implementation and by 28.2% from baseline to mHealth implementation (*P*<.001). From baseline, patient report of provider inquiry regarding medication complications increased in the guidelines and mHealth phases by 12.9% and 59.6%, respectively, (*P*<.001). The proportion of patients reporting that providers asked other questions relevant to their illness increased from baseline through guidelines implementation by 27.8% and to mHealth implementation by 66.3% (*P*<.001). Follow-up scheduling increased from baseline to the guidelines phase by 20.6% and the mHealth phase by 39.8% (*P*<.001).

**Conclusions:**

Results from this study of an mHealth app in 10 PHC facilities in Lebanon indicate that the app has potential to improve adherence to guidelines and quality of care. Further studies are necessary to determine the effects of patient-controlled health record apps on provider adherence to treatment guidelines, as well as patients’ long-term medication and treatment adherence and disease control.

## Introduction

An estimated 4.8 million Syrians have fled the conflict to neighboring countries and are registered or awaiting registration with the United Nations High Commissioner for Refugees (UNHCR), in addition to a population of unregistered refugees unknown in number [1]. As of January 2017, over one million Syrian refugees were registered with UNHCR in Lebanon [1]. With an estimated 183 refugees per 1000 inhabitants at the end of 2015, Lebanon hosts the highest ratio of refugees-to-host population worldwide [2]. The humanitarian response in Lebanon is coordinated through an interagency mechanism established by UNHCR and the Lebanese government, integrating refugee assistance into existing clinics. Delivery of health services for Syrian refugees is based on a primary health care (PHC) strategy. Syrian refugees can utilize primary health care services paying subsidized rates at designated existing primary health care centers and primary level facilities across Lebanon, unless they choose to seek care at private clinics [3,4]. Delivery of noncommunicable disease (NCD) treatment for Syrian refugees and vulnerable Lebanese not seeking care in the private sector is based on routine care in primary health facilities with referral to secondary and tertiary care for specialist management.

Both Lebanese and Syrian populations are in the late stages of the epidemiologic transition from communicable, maternal, neonatal, and nutritional conditions to NCDs. In Lebanon, both the host community and refugee populations suffer from high NCD burdens [5,6]. Type 2 diabetes prevalence has been estimated at 7.4% in Syria and 14.4% in Lebanon [7]. Previous reports have estimated regional prevalence of hypertension at 29.5% in Syria and for Lebanon variously at 24.9% and 28.8% [8-10]. Ischemic heart disease and stroke, for which hypertension and diabetes have substantially increased risk, are the leading causes of death in Lebanon and aside from conflict-related death, in Syria as well [11,12]. Moreover, based on the 2012 age-specific mortality risks throughout their lifetime, the probability of an individual aged between 30 and 70 years dying from cancer, cardiovascular disease, chronic respiratory disease, or diabetes is 12% in Lebanon and 19% in Syria; figures matched only by risks of conflict-related death in Syria [11,12]. Management of NCDs can be difficult and requires continuity of care, which is difficult for refugees and poses challenges to health services and systems. The burden placed on Lebanon’s highly fragmented and privatized health system by refugee influx is immense though not unique in the new global displacement environment [13]. Increasingly, displaced populations are urban and from low- and middle-income countries where NCDs constitute a significant burden of disease. Not only the numbers but also the complexity of conditions pose challenges to health systems addressing the needs of both refugee and hosts with NCDs. The practice pattern in which persons with even mild hypertension are seen by cardiologists and persons with well-controlled mild diabetes consult endocrinologists rather than primary care physicians increases the complexity and costs of care. Limited resource availability has prioritized care to PHC conditions, limiting more expensive specialist care [3,14,15]. We undertook a study to evaluate the effectiveness of treatment guidelines and an mHealth app on quality of care and health outcomes in primary care settings.

## Methods

### Study Design

A longitudinal cohort study was implemented from January 2015 to August 2016 in primary health facilities in Lebanon that serve both Syrian refugees and Lebanese. Its two research aims were (1) to develop, adapt, and test existing standards and guidelines for treatment, including counseling, of persons with hypertension and type 2 diabetes (or both) and (2) to evaluate the effectiveness of an mHealth tool. Standard best-practice guidelines were adapted to the local context using national protocols, prescribing practices, and the primary care context where they would be applied. [16-18]. Providers were subsequently trained on guidelines and provided with written materials to support clinical decision making. The mHealth app included a personally controlled health record (PCHR), informational printouts for patients on prescriptions, and lifestyle behaviors and served as an electronic medical record and decision support tool for providers. If patients move locations without their medical records, key diagnostic and treatment elements are available from the patient’s cell phone subscriber identity module card, which constitutes the PCHR. The mHealth tool has the potential to improve quality and continuity of care, health literacy, mobility of medical records, and health outcomes for patients. Providers were trained in use of the app, and support was provided to health facilities for its implementation [19]. The study used a phased introduction of the two interventions over 20 months with longitudinal measurement of outcomes.

### Study Participants

**Figure 1:**
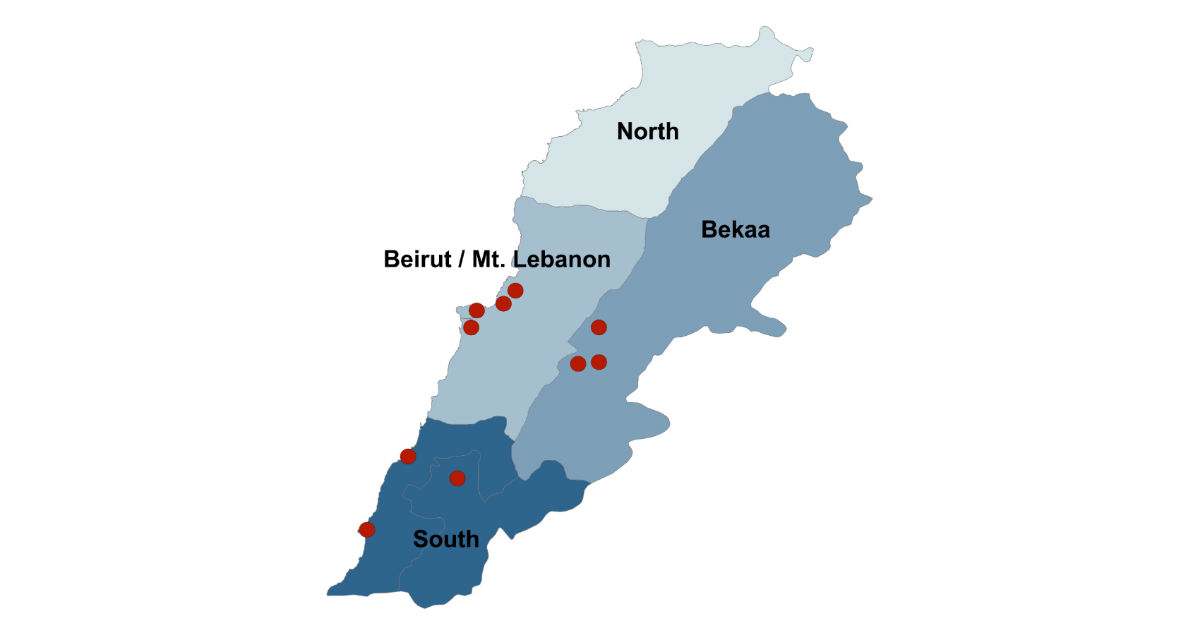
Participating primary health centers.

Participants consisted of patients at 10 health care centers in Lebanon supported by the International Organization for Migration or the International Medical Corps in the South (n=3), Bekaa (n=3), and Beirut and Mount Lebanon (n=4) governorates (Figure 1). Patients at these locations were predominantly Lebanese and Syrian refugees. Individuals without a diagnosis of hypertension or type 2 diabetes, those aged less than 40 years, and adults lacking capacity to independently participate in interviews were excluded.

A total of 1020 participants were enrolled and 793 (77.75%) completed the study. Sample size calculations were based on the estimated proportion of providers adhering to treatment guidelines, with an assumed baseline rate of 50% for adherence to guidelines (the most conservative rate that would ensure the ability to detect significant differences from all other rates). This is a reasonable assumption given that proposed guidelines did not differ substantially from other best practice guidelines; thus, patients being enrolled at baseline could already be on recommended treatment. Sample size calculations were performed using Stata 13 (StataCorp LLC), assumed alpha=.05 and beta=.20 (power=0.80), and were one-sided based on the assumption that quality of care will not decrease because of the intervention. The final sample of 793 participants was sufficient to detect increases ≥5.0% for provider adherence to guidelines.

### Study Procedures and Outcome Measures

This study was designed using a mixed-methods approach with qualitative and quantitative data collected throughout. Patients were recruited at clinics, and if they indicated willingness to participate, a follow-up phone call was made. This verified consent, and a baseline interview collected information on demographic characteristics; medical history and recent care-seeking behaviors; and knowledge, attitudes, and practices related to type 2 diabetes and hypertension. Following enrollment, medical record reviews were also conducted for each patient, recording information related to provider compliance with guidelines and quality of care. Additional information was collected on frequency of clinic visits, patient status (death and loss to follow-up), and disease-specific patient outcomes (complications and adverse events of hypertension and type 2 diabetes). Data from phone interviews and record reviews were collected at the end of each study phase (guidelines and mHealth). In addition, a subset of patients visiting study facilities during the course of the study were telephoned within 10 days of their visit to complete a brief exit interview.

### Clinical Measurements

Clinical measurements including height, weight, blood pressure (BP), glycated hemoglobin (HbA1c), fasting blood sugar, and random blood sugar were extracted from patient records at baseline, following implementation of the treatment guidelines, and after implementation of the PCHR. At the end of the mHealth intervention study phase, clinical measurements were also extracted from the PCHR database to triangulate facilities’ record keeping with data entered in the PCHR by providers.

### Patient-Provider Interaction

The quality of patient-provider clinical interactions was assessed based on patient reports from exit interviews conducted during each study phase with a subset of patients that visited a study facility. As with clinical measurements, data from the PCHR was used to compare patient report of clinical interactions with that reported by providers in the app. Interactions were evaluated based on four key indicators of providers’ compliance with treatment guidelines: (1) provider inquiry of medical history, (2) query about complications with prescribed medication, (3) prompting for questions from the patient, and (4) recommending follow-up or referral care. Additionally, clinical interactions were evaluated based on report of lifestyle counseling on smoking, alcohol consumption, physical activity, and dietary patterns.

### Medication Prescription and Use

Medication prescription and use were assessed and compared using data obtained both through patient self-report during phone interviews conducted in each study phase, as well as documentation in patients’ health facility records.

### Analysis

**Figure 2:**
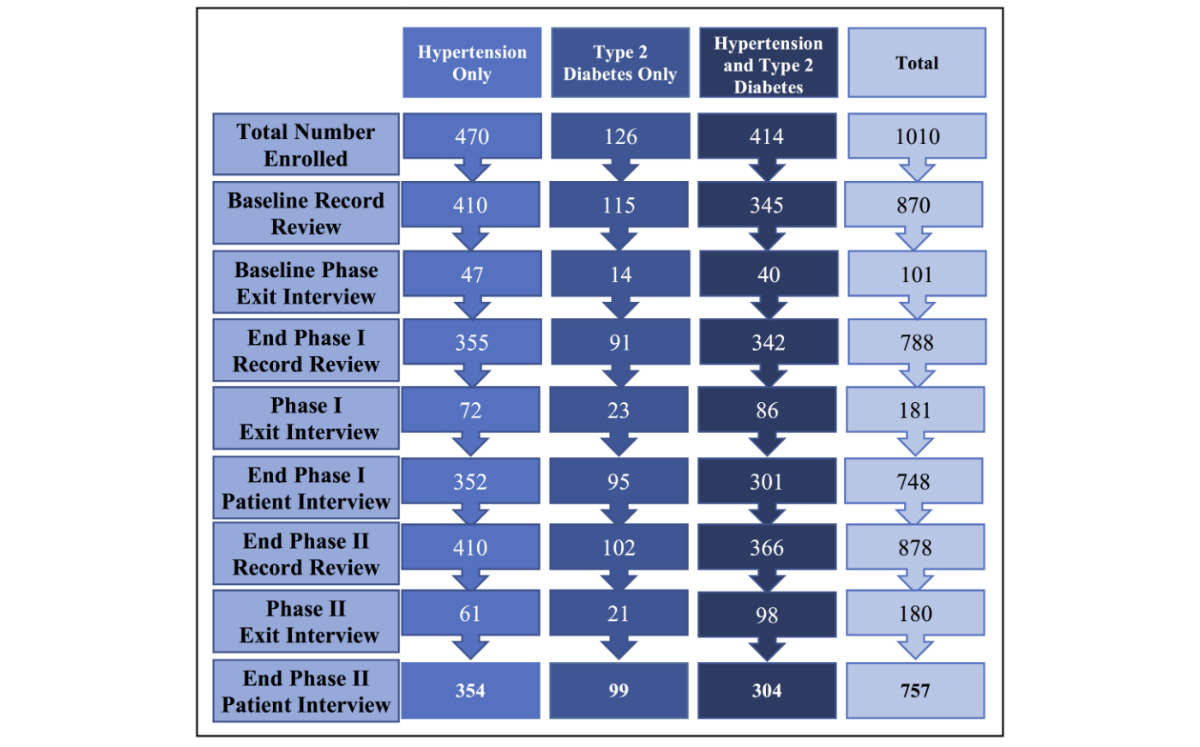
Patient follow-up and response rates.

Data were collected with tablets using the Magpi mobile data platform by DataDyne LLC (Washington, DC) and analyzed using Stata 13 (College Station, TX) using descriptive statistics and standard methods for comparison of means and proportions. BP readings monitored control among hypertensive patients, and the HbA1c test was the preferred measure for classifying type 2 diabetic patients; when not available, random or fasting blood sugar was used [20,21]. A sequenced process-based classification used patient records, clinical data, and prescriptions to assign a uniform diagnosis category to patients in cases where reporting was inconsistent over time. A total of 8 patients remained with an unclassified diagnosis and were subsequently dropped from final analysis to ensure reliable reporting by condition. Utilization of the mHealth app by practitioners was low. A total of 154 records were extracted from the app dataset, whereas a total of 878 record reviews and 761 patient interviews were completed in the mHealth phase (Figure 2). Differences in patient characteristics and condition control status were examined using chi-square and *t* test methods. An immediate form of two-sample tests of proportions was performed using the Stata *prtesti* command to determine whether the proportions in the mHealth app and paper records were statistically different.

This study was approved by the ministry of public health in Lebanon and the institutional review board at the Johns Hopkins Bloomberg School of Public Health.

## Results

### Clinical Measurements

Clinical measurements extracted from patient records and hypertension and type 2 diabetes control data are presented in Table 1. Compared with baseline data, significant declines in reporting of BP (−11.4%, *P*<.001) and blood sugar (−6.9%, *P*=.03) measurements were observed following implementation of treatment guidelines. Recording of systolic and diastolic BP measurements also declined after the mHealth phase as compared with baseline (−8.4%, *P*=.001); however, body mass index (BMI) reporting increased at the end of the mHealth phase from both baseline (8.1%, *P*<.001) and the end of the guidelines phase (7.7%, *P*<.001). Baseline clinical test results included all information in clinic records, regardless of when it was reported; because reporting is not time bound, changes in completeness of reporting are difficult to interpret because values could have been reported at one of a number of prior visits. Changes in clinical measurements and the control of hypertension and type 2 diabetes were not significant in the mHealth phase, likely because of short implementation time and challenges with provider uptake of the app.

**Table 1:** Patient biometric health measures from the noncommunicable disease (NCD) guidelines and mobile health (mHealth) records for refugees in the Lebanon study.

Parameter		Baseline		Phase I^a^		Phase II^b^		Change by phase		
		n (%)	95% CI	n (%)	95% CI	n (%)	95% CI	Phase I versus baseline	Phase II versus baseline	Phase II versus phase I
								*P* value	*P* value	*P* value
**Body mass index (BMI)**		**N=870**		**N=789**		**N=878**				
	Total patients with BMI measured	67 (7.7)	6.0-9.7	64 (8.1)	6.3-10.2	139 (15.8)	13.5-18.4	.76	<.001	<.001
		**N=67**		**N=64**		**N=139**				
	Median	32.8		33		31.5				
	Mean	33.5	31.9-35.1	34	32.3-35.8	32.1	31.1-33.1	.65	.13	.04
	BMI (normal)^c^	5 (8)	2.5-16.6	5 (8)	2.6-17.3	12 (8.6)	4.5-14.6	.94	.78	.85
	BMI (overweight)^d^	15 (22)	13.1-34.2	14 (22)	12.5-34.0	42 (30.2)	22.7-38.6	.94	.24	.22
	BMI (obese)^e^	47 (70)	57.7-80.7	45 (70)	57.6-81.1	85 (61.2)	52.5-69.3	.98	.21	.21
**Hypertension**		**N=755**		**N=697**		**N=776**				
	Total hypertension patients with blood pressure measured	371 (49.1)	45.5-52.8	263 (37.7)	34.1-41.4	316 (40.7)	37.2-44.3	<.001	.001	.24
**Blood pressure**		**N=371**		**N=263**		**N=316**				
	Controlled blood pressure (BP)^f^	238 (64.2)	59.0-69.0	183 (69.6)	63.6-75.1	223 (70.6)	65.2-75.5	.15	.08	.80
	Uncontrolled systolic BP^g^	81 (21.8)	17.7-26.4	42 (16.0)	11.8-21.0	59 (18.7)	14.5-23.4	.07	.31	.40
	Uncontrolled diastolic BP^h^	7 (1.9)	0.8-3.8	5 (1.9)	0.6-4.4	6 (1.9)	0.7-4.1	.99	.99	.99
	Uncontrolled BP^i^	45 (12.1)	9.0-15.9	33 (12.5)	8.8-17.2	28 (8.9)	6.0-12.6	.88	.17	.15
**Diabetes**		**N=460**		**N=433**		**N=468**				
	Diabetes patients with blood test results^j^	173 (37.6)	33.2-42.2	133 (30.7)	26.4-35.3	159 34.0)	29.7-38.5	.03	.25	.30
**Diabetes control^k^**		**N=173**		**N=133**		**N=159**				
	Controlled	78 (45.1)	37.5-52.8	56 (42.1)	33.6-51.0	83 (52.2)	44.1-60.2	.60	.20	.09
	Uncontrolled	95 (54.9)	47.2-62.5	77 (57.9)	49.0-66.4	76 (47.8)	39.8-55.9			

^a^Guideline implementation.

^b^mHealth implementation.

^c^BMI<25 kg/m^2^ normal.

^d^BMI>25kg/m^2^ overweight.

^e^BMI>30kg/m^2^ obese.

^f^Controlled: BP<140/90.

^g^Uncontrolled: Systolic BP>140 (Diastolic BP<90).

^h^Uncontrolled: Diastolic BP>90 (Systolic BP<140).

^i^Uncontrolled: BP>140/90.

^j^Includes HbA1c, FBS, RBS, or any combination of those tests.

^k^Based on results from either HbA1c, FBS, or RBS; if multiple tests available preference is given first to HbA1c (controlled defined as <7.0%), then FBS (controlled defined as <120mg/dL), then RBS (controlled defined as <100 mg/dL).

**Figure 3:**
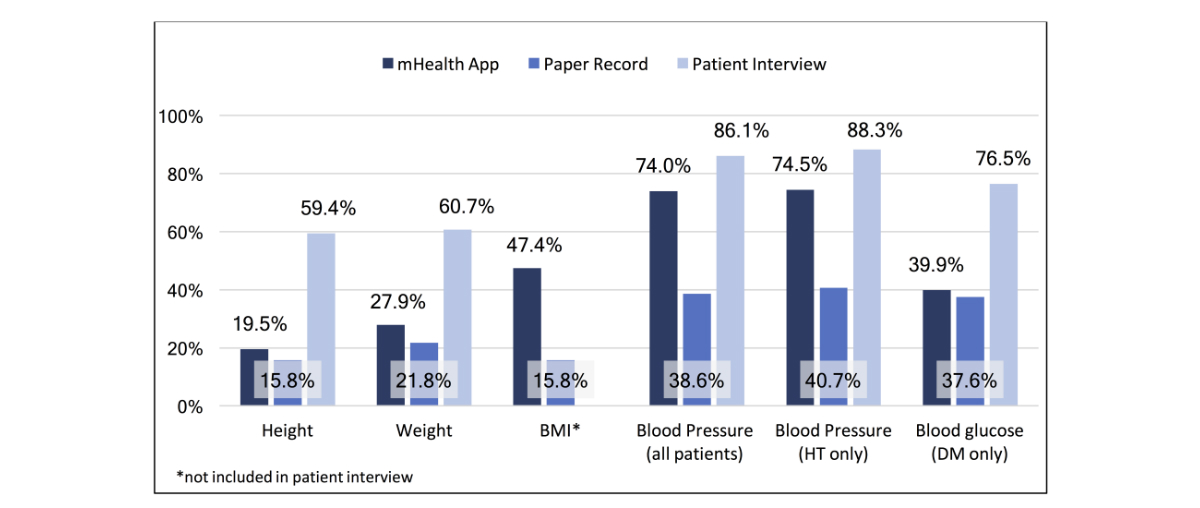
Clinical iIndicator measurement by reporting source.

Comparison of data reported in the mHealth app with paper records and patient interviews during app implementation is presented in Figure 3. Comparing information reported in paper records following implementation of the mHealth app with data extracted directly from the app, BP measures were reported for a substantially larger proportion of patients in the app (114/154, 74.0% patients with app data vs 339/878, 38.6% patients with paper records, *P*<.001). Similarly, reporting of weight, height, and BMI were all more frequently reported with the app than with paper records as follows: weight, 43/154 (28%) patients from app data versus 191/878 (21.8%) from paper records, *P*=.10; height, 30/154 (19%) patients from the app versus 139/878 (15.8%) from paper records, *P*=.25; BMI, 73/154 (47%) patients from the app versus 139/878 (15.8%) from paper records, *P*<.001. Among hypertensives, BP readings were more commonly reported with the app than with paper records (114/153, 75% patients from the app vs 776/878, 40.7% patients from paper records; *P*=.24). Among type 2 diabetics, blood sugar tests were reported for a slightly larger proportion of patients with the app than with patient records (61/153, 39.9% of patients from app data vs 159/468, 34.0% from paper records; *P*=.19). Higher reporting by clinicians using the app supports the likelihood that mixed results regarding changes in provider adherence to guidelines measured following the mHealth phase are because of poor reporting with paper records more than poor performance of the app. Furthermore, over twice as many patients reported that measurement of weight, height, BP, and blood glucose had been taken than the mHealth app and/or paper records showed, suggesting that care quality may be better in actuality than as reflected by completeness of reporting measures.

### Patient-Provider Interaction

Statistically significant increases were detected in all four measures of patient-provider clinical interactions (Table 2). The proportion of patients reporting that the provider took a medical history during the enrollment phase (72/101 patients, 71.3%) increased by 16.6% to 87.9% (160/182) patients in the guideline phase and by 28.2% from enrollment to 99.4% (179/180) patients in the mHealth phase (*P*<.001). Just over a third (36/100, 36%) of patients reported that the provider asked about medication complications at the most recent care visit during the enrollment phase. In the guidelines and mHealth phases, this increased from enrollment by 12.9% to 48.9% (89/182) patients and by 59.6% to 95.6% (172/180) patients, respectively (change from enrollment to guidelines phase *P*=.04; change from enrollment to mHealth phase, *P*<.001). The proportion of patients reporting that providers asked other questions relevant to their illness increased from 32.0% (32/100) patients during the enrollment phase to 59.8% (107/179) patients during the guidelines phase and 98.3% (177/180) patients in the mHealth phase (respective increases of 27.8% and 66.3%, *P*<.001). A significantly higher proportion of patients also reported providers scheduling a follow-up appointment or being referred for specialty care, from 58.0% (58/100) patients in the enrollment phase to 78.6% (143/182) patients in the guidelines phase and 97.8% (176/180) patients in the mHealth phase (respective increases of 20.6% and 39.8%, *P*<.001).

Patient report of provider counseling about lifestyle behaviors such as smoking, alcohol consumption, physical activity, and dietary patterns also improved (Table 2). However, the provider’s reports of counseling carried out significantly differed in patient records and the mHealth app. Data extracted from the mHealth app showed a much higher proportion of providers offering lifestyle counseling as compared with notations in patient records. Smoking cessation counseling was reported for 16.9% (26/154) patients from the app data versus 11.4% (96/844) patients from paper records (*P*=.06). Much larger differences were observed in health dietary habit counseling (119/154, 77.3% patients from app data vs 77/878, 8.8% from paper records; *P*<.001) and physical activity counseling (92/154, 59.7% patients from app data vs 62/878, 7.1% from paper records; *P*<.001).

**Table 2:** Quality of interaction with providers reported by patients in the noncommunicable disease (NCD) guidelines and mobile health (mHealth) records for refugees in Lebanon study. Data reported by patients in exit interviews were conducted via phone.

Parameter		Baseline (N=101)		Phase I^a^ (N=181)			Phase II^b^ (N=180)			Change comparison					
		n (%)	95% CI		n (%)	95% CI		n (%)	95% CI		Phase I versus baseline		Phase II versus baseline		Phase II versus phase I	
										*P* value		*P* value		*P* value	
**Provider interaction**															
	Asked about medical history	72 (71.3)	61.4-79.9		160 (87.9)	82.3-92.3		179 (99.4)	96.9-100		<.001		<.001		<.001
	Asked about complications with medications	36 (36.0)	26.6-46.2		89 (48.9)	41.4-56.4		172 (95.6)	91.4-98.1		.04		<.001		<.001
	Asked other questions	32 (32.0)	23.0-42.1		107 (59.8)	52.2-67.0		177 (98.3)	95.2-99.7		<.001		<.001		<.001
	Provided follow-up appointment or referral	58 (58.0)	47.7-67.8		143 (78.6)	71.9-84.3		176 (97.8)	94.4-99.4		<.001		<.001		<.001
**Lifestyle counseling received**															
	Quit or stop using tobacco	32 (31.7)	22.8-41.7		79 (44.1)	36.7-51.7		157 (87.2)	81.4-91.7		.04		<.001		<.001
	Reduce salt consumption	56 (55.4)	45.2-65.3		147 (82.1)	75.7-87.4		172 (95.6)	91.4-98.1		<.001		<.001		<.001
	Fruit and vegetable consumption	48 (47.5)	37.5-57.7		141 (78.8)	72.0-84.5		172 (95.6)	91.4-98.1		<.001		<.001		<.001
	Reduce fat consumption	56 (55.4)	45.2-65.3		150 (83.8)	77.6-88.9		172 (95.6)	91.4-98.1		<.001		<.001		<.001
	Engage in physical activity	43 (43.0)	33.1-53.3		138 (77.1)	70.2-83.0		167 (92.8)	88.0-96.1		<.001		<.001		<.001
	Lose weight	31 (31.0)	22.1-41.0		120 (67.0)	59.6-73.9		155 (86.1)	80.2-90.8		<.001		<.001		<.001

^a^Guideline implementation.

^b^mHealth implementation.

### Medication Prescription and Use

**Figure 4:**
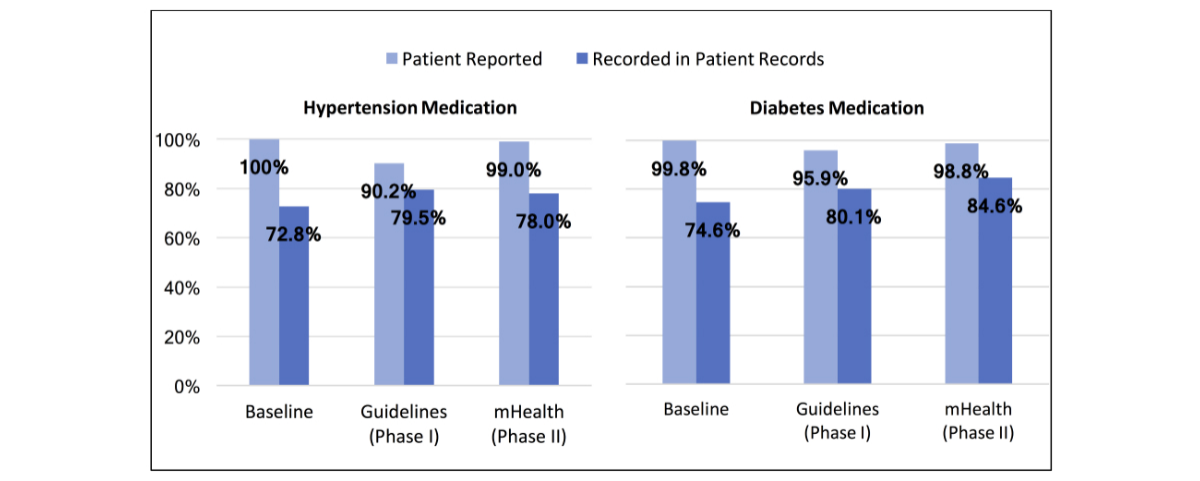
Medication prescription by reporting method.

Medication compliance and other compliance variables were also used to evaluate performance and outcomes related to guideline training and app adoption (Table 3). The proportion of patients reporting receiving prescriptions of medication for hypertension and type 2 diabetes was consistently high, exceeding 90% at baseline and in both study phases (Figure 4). On the basis of reporting in patient records, there was a small but significant increase in patients prescribed medication for hypertension from baseline to the end of the guidelines phase (6.6% increase, *P*=.003) and from baseline to the end of the mHealth phase (5.1% increase, *P*=.02). Unlike notations in patient records, the proportion of patients self-reporting being prescribed hypertension medication decreased significantly from baseline to the end of the guidelines phase (9.8% decrease, *P*<.001); however, this proportion significantly increased in the mHealth phase by 8.9% (*P*<.001). The proportion of patients self-reporting current use of hypertensive medications decreased significantly from baseline to the end of the guidelines phase (3.9% decrease, *P*<.001) and from baseline to the end of the mHealth phase (2.3% decrease, *P*=.02).

Among patients with type 2 diabetes, there was a significant increase in medication prescription in patient records from baseline to the end of the guidelines phase (5.6% increase, *P*=.047) and from baseline to the end of the mHealth phase (10.1% increase, *P*<.001). Unlike in patient records, the proportion of patients self-reporting being prescribed medication decreased significantly from baseline to the end of the guidelines phase (3.9% decrease, *P*<.001); however, this proportion significantly increased in the mHealth phase by 2.9% (*P*=.03). The proportion of type 2 diabetics reporting current diabetes medication use decreased significantly from baseline to the end of the guidelines phase (6.3% decrease, *P*<.001) and from baseline to the end of the mHealth phase (3.8% decrease, *P*=.02).

Overall, medication compliance was good among both hypertensive and type 2 diabetic patients across follow-up. The proportion of hypertensive patients reporting they had stopping prescribed medication for 2 weeks or longer in the 3 months preceding interview was highest at baseline (70/755, 9.3%) and lowest at the end of the guidelines phase (49/604, 8.1%). Interruptions in diabetes medication was highest at baseline and at the end of the guidelines phase (38/506, 7.5% and 29/383, 7.6%, respectively) and lowest at the end of the mHealth phase (14/256, 5.5%). Observed changes in interruption of medication for hypertension or diabetes were not significant among any of the study phases. Reasons for stopping medication were similar by condition and across the study periods. Cost was the primary reason for stopping medication (62.9%-74.4%, depending on the condition and study phase). The other common reasons were advice from the provider and the patient perception that that their condition had improved, which is particularly challenging as, given that hypertension and diabetes have few if any symptoms, patients likely ascribe symptoms to their disease that are not related.

**Table 3:** Medication and compliance among patients in the noncommunicable disease (NCD) guidelines and mobile health (mHealth) records for refugees in Lebanon study.

Parameter			Baseline		Phase I^a^		Phase II^b^		Change comparison		
			n (%)	95% CI	n (%)	95% CI	n (%)	95% CI	Phase I versus baseline	Phase II versus baseline	Phase II versus phase I
									*P* value	*P* value	*P* value
**Hypertension medication**			**N=755**		**N=697**		**N=776**				
	All hypertension patients prescribed medication for hypertension^c^		550 (72.8)	69.5-76.0	554 (79.5)	76.3-82.4	605 (78.0)	74.9-80.8	.003	.02	.48
	Not reported in patient record^c^		43 (5.7)	4.2-7.6	92 (13.2)	10.8-15.9	35 (4.5)	3.2-6.2	<.001	.29	<.001
**Self-reported hypertension medication**			**N=873**		**N=652**		**N=418**				
	Ever prescribed medication		873 (100)	99.5-100	588 (90.2)	87.6-92.4	414 (99.0)	97.6-99.7	<.001	.004	<.001
			**N=754**		**N=605**		**N=413**				
	Currently taking hypertension medication^d^		740 (98.1)	96.9-99.0	570 (94.2)	92.0-95.9	396 (95.9)	93.5-97.6	<.001	.02	.24
	Stopped taking medicines for 2+ weeks in the past 3 months^d^		70 (9.3)	7.3-11.6	49 (8.1)	6.1-10.6	35 (8.6)	6.0-11.7	.45	.69	.80
**Noncompliance^e^**			**N=76**		**N=50**		**N=36**				
	**When medication was stopped**										
		Stopped taking in Syria	9 (11.8)	5.6-21.3	10 (20.0)	10.0-33.7	5 (13.9)	4.7-29.5	.21	.76	.47
		Taking in Syria, stopped in Lebanon	36 (47.4)	35.8-59.2	13 (26.0)	14.6-40.3	15 (41.7)	25.5-59.2	.02	.58	.13
		Started taking in Lebanon but stopped	31 (40.8)	29.6-52.7	27 (54.0)	39.3-68.2	16 (44.4)	27.9-61.9	.15	.72	.39
**Diabetes medication**			**N=460**		**N=433**		**N=468**				
	% of all diabetes patients prescribed medication for diabetes^c^		343 (74.6)	70.3-78.5	347 (80.1)	76.1-83.8	396 (84.6)	81.0-87.8	.047	<.001	.08
	Not reported in patient record^c^		28 (6.1)	4.1-8.7	54 (12.5)	9.5-16.0	16 (3.4)	2.0-5.5	.001	.06	<.001
**Self-reported diabetes medication**			**N=537**		**N=394**		**N=260**				
	Ever prescribed medication		536 (99.8)	98.9-100	378 (95.9)	93.5-97.7	257 (98.8)	96.7-99.8	<.001	.07	.03
			**N=506**		**N=384**		**N=259**				
	Currently taking diabetes medication^d^		488 (96.4)	94.4-97.9	346 (90.1)	86.7-92.9	240 (92.7)	88.8-95.5	<.001	.02	.26
	Stopped taking medicines for 2+ weeks in the past 3 months^d^		38 (7.5)	5.4-10.2	29 (7.6)	5.1-10.7	14 (5.5)	3.0-9.0	.97	.29	.30
**Noncompliance^e^**			**N=43**		**N=35**		**N=15**				
	**When medication was stopped**										
		Stopped taking in Syria	6 (14.0)	5.3-27.9	7 (20.0)	8.4-36.9	5 (33.3)	11.8-61.6	.48	.10	.32
		Taking in Syria, stopped in Lebanon	22 (51.2)	35.5-66.7	9 (25.7)	12.5-43.3	3 (20.0)	4.3-48.1	.02	.04	.67
		Started taking in Lebanon but stopped	15 (34.9)	21.0-50.9	19 (54.3)	36.6-71.2	7 (46.7)	21.3-73.4	.09	.43	.63

^a^Guideline implementation.

^b^mHealth implementation.

^c^As reported in patient health records.

^d^Among those prescribed medication.

^e^Among only patients that stopped taking medication in the past 3 months.

## Discussion

### Principal Findings

Consistent and complete reporting is essential to monitor changes and trends in clinical measurements for diabetic and hypertensive patients. Notwithstanding relatively low provider uptake of the app, reporting of nearly all clinical measures was improved when the provider used the app rather than written patient records. Data extracted from the mHealth app also showed a greater proportion of providers offering lifestyle counseling as compared with counseling reported in paper medical records kept by health facilities, and statistically significant improvements were observed in all four measures of patient-provider interaction. This clearly demonstrated the advantages of electronic reporting and the potential for mHealth apps to improve quality of clinical care for chronic NCDs. Despite the possible benefits of mHealth for improving case management of hypertension and type 2 diabetes, there were difficulties in developing and deploying new technologies that diminished the utilization and potential benefits of the mHealth app.

The challenges in this study are neither unique to the project nor to the context. Data quality in electronic health records (EHRs), particularly completeness of reporting, is indispensable for the associated decision support components to prove effective. Poor reporting observed in this project were similarly observed in a recent trial incorporating the Screening Tool of Older People’s Prescriptions prescribing criteria in a primary care EHR, which demonstrated the need for continued assessment of data quality and improvement on potentially inappropriate prescription rates in community primary care settings [22]. A 2013 study of implementation of national guidelines and an associated structured type 2 diabetes and hypertension patient record reported poor use of structured record by providers as a primary explanation for null benefit of the intervention [23]. Other barriers previously documented include cost, language, literacy, availability or connectivity issues, and perceived increase in workload; connectivity, language, and workload increase presented challenges in this study [24,25]. The aim of this project focused on both PCHR app development and pilot evaluation of the app. However, allowing a longer time period to develop and test the app, followed by a subsequent pilot test, would have been a more appropriate design if time permitted. As such, mixed findings on the results of the mHealth app use should not minimize the consideration of the app’s potential effectiveness.

The nature of mHealth interventions vary widely, ranging from health promotion and disease surveillance to remote monitoring, care support, and decision support tools [26]. Previous research has primarily focused on remote monitoring and care support tools, and while there is a considerable body of literature on the design and implementation of personal health records, considerably less evidence is available for decision support functions, particularly in low- and middle-income countries [26,27]. EHRs have previously shown effectiveness in improving type 2 diabetic patient health outcomes and clinical practice in developed countries but do not adequately capture the potential added benefits of provider decision support elements as were incorporated in the PCHR app developed for this study [28]. The PCHR app developed for this study might lead to better uptake and prove more effective in other settings, with providers more open to newer technology, with fewer reporting requirements, without electronic information management systems, and where providers were more open to changing their clinical practice behaviors. The patient-controlled portability component may also improve patient knowledge of their condition and continuity of care, in particular in the context of migration, two outcomes which were not assessed in this study.

### Limitations

Comparison of completeness of reporting across study phases may have underestimated changes in patient and provider practices in the guidelines and mHealth phases where all available information in the patient record was included at baseline, regardless of what was recorded at the most recent visit. Simultaneous development and introduction of the app led to frustration among users when the app did not perform as expected, requiring frequent software updates, which reduced provider enthusiasm. Another barrier to uptake was multiple reporting requirements and electronic record systems, which led to the perception that the app was redundant (despite dissimilarities to existing systems in most cases). Finally, including patients and providers from only 10 health facilities limits representativeness of findings, and the results may not be generalizable to elsewhere in Lebanon or other settings.

### Conclusions

The mHealth app was successful in improving some quality of care indicators, indicating there is potential for clinical decision making support tools to enhance capacity for NCD care in PHC centers. Recording rates of BMI improved with use of the mHealth app; however, there was a decline in the recording of BP and blood sugar levels; recording rates for all three measures were higher in the mHealth app than in paper records. Patient-provider interactions, life style counseling, and scheduling of follow-up appointments improved with use of the mHealth app, suggesting there were some improvements in quality of care. Only small improvements in the proportion of patients with controlled hypertension and diabetes were observed between baseline and the end of the mHealth phase, and these differences were not statistically significant.

Results from this study of an mHealth app in 10 PHC facilities in Lebanon indicate the app has potential to improve adherence to guidelines and quality of care. Greater support to service providers during the adoption of the apps, customization of the apps for specific settings, and longer follow-up periods may aid in better characterizing possible benefits of this and other mHealth apps for NCD management. Further studies are necessary to determine the effects of this and similar PCHR apps on provider adherence to treatment guidelines, as well as patients’ long-term medication and treatment adherence and disease control. Additional testing in less developed settings, including both rural locations and emergency contexts, will help provide evidence on the potential of these apps and factors associated with uptake and effectiveness across a broader range of contexts. Expanding the evidence on mHealth apps so that it is sufficient to inform decision making on adoption of these tools is essential, given their potential benefits.

## Abbreviations

BMI: body mass index
BP: blood pressure
EHR: electronic health record
HbA1c: glycated hemoglobin
mHealth: mobile health
NCD: noncommunicable disease
PCHR: personally controlled health record
PHC: primary health care
UNHCR: United Nations High Commissioner for Refugees
